# Persons Living With Primary Immunodeficiency Act as Citizen Scientists and Launch Prospective Cohort Body Temperature Study

**DOI:** 10.2196/22297

**Published:** 2020-11-30

**Authors:** Shouling Zhang, Tiffany S Henderson, Christopher Scalchunes, Kathleen E Sullivan, Artemio M Jongco III

**Affiliations:** 1 Division of Allergy and Immunology Icahn School of Medicine at Mount Sinai New York, NY United States; 2 Department of Pediatrics Cohen Children’s Medical Center Donald and Barbara Zucker School of Medicine at Hofstra/Northwell New Hyde Park, NY United States; 3 Immune Deficiency Foundation Towson, MD United States; 4 Division of Allergy and Immunology Children's Hospital of Philadelphia Philadelphia, PA United States; 5 Division of Allergy and Immunology Donald and Barbara Zucker School of Medicine at Hofstra/Northwell Great Neck, NY United States; 6 Center for Health Innovations and Outcomes Research Feinstein Institute for Medical Research Manhasset, NY United States

**Keywords:** fever, temperature, primary immunodeficiency

## Abstract

**Background:**

Although fever is considered a sign of infection, many individuals with primary immunodeficiency (PI) anecdotally report a lower-than-normal average body temperature on online forums sponsored by the Immune Deficiency Foundation (IDF). There is limited knowledge about the average body temperature and fever response in PI.

**Objective:**

This study aims to compare median body temperatures between adults with and without PI diagnoses living in the same household and to engage individuals living with PI throughout the research process.

**Methods:**

Patients with PI designed and launched a prospective cohort comparison study as citizen scientists. A multidisciplinary team designed and implemented a patient-informed study with continuous patient-driven input. Median body temperatures were compared between the 2 cohorts using the Mann-Whitney test with Bonferroni correction. The IDF conducted a post-study patient experience survey.

**Results:**

Data from 254 households were analyzed (254/350, 72.6% participation rate). The PI population was predominantly female (218/254, 85.8%), White (248/254, 97.6%), and with a median age of 49 years. The non-PI population was largely male (170/254, 66.9%), White (236/254, 92.9%), and with a median age of 53 years. Common variable immunodeficiency was the most common PI diagnosis (190/254, 74.8%). Of the 254 individuals with PI, 123 (48.4%) reported a lower-than-normal nonsick body temperature, whereas 108 (42.5%) reported a normal (between 97°F and 99°F) nonsick body temperature. Among individuals with PI, when infected, 67.7% (172/254) reported the absence of fever, whereas 19.7% (50/254) reported a normal fever response. The recorded median body temperature was minimally but statistically significantly higher for patients with PI in the morning. Although 22.4% (57/254) of patients with PI self-reported illness, a fever of 100.4°F or higher was uncommon; 77.2% (196/254) had a normal temperature (between 97°F and 99°F), and 16.2% (41/254) had a lower-than-normal temperature (between 95.0°F and 96.9°F) when sick. For these sick patients with PI, the median body temperature was minimally but statistically significantly higher for patients in the morning and early evening. Overall, 90.9% (231/254) of participants would be very likely to participate in future IDF studies, although 94.1% (239/254) participants had never taken part in previous studies.

**Conclusions:**

To our knowledge, this is the first study to evaluate average body temperature in individuals with PI. Although there were small statistically significant differences in body temperatures between PI and non-PI subjects, the clinical significance is unclear and should be interpreted with caution, given the methodological issues associated with our small convenience sample and study design. As PIs are heterogeneous, more research is needed about how the fever response differs among diverse PIs compared with healthy controls. This study highlights that individuals with PI are knowledgeable about their health and can offer unique insights and direction to researchers and clinicians.

## Introduction

### Background

Primary immunodeficiency (PI) diseases represent a class of approximately 450 rare, genetic, and chronic disorders in which there is a defect in the human immune system [1]. To function properly, an immune system must detect and protect against a wide variety of pathogens. It must distinguish between foreign pathogens and their own cells. When any component is absent or dysfunctional, the result is a susceptibility to severe, persistent, unusual, and recurrent infections [1,2].

Normal body temperature is considered an oral measurement of approximately 98.6°F (37°C). Fluctuations in body temperature of 1°F (0.6°C) are known to occur throughout the day depending on the activity level and the time of day. This normal temperature was established in the 19th century; however, more recent studies suggest a lower body temperature [3]. Fever is a proinflammatory response that involves cytokine release, which may include tumor necrosis factor and interleukin-1 [4]. Fever is considered the immune system’s response to pathogens to make the body a less favorable environment for infection.

At present, there is a dearth of literature on the average body temperature in persons with PI, and more information on the fever response in PI is needed. Fever is often considered the first sign of infection. Some, but not all, patients with PI can be deficient in generating cytokine responses that may also contribute to pyrogen release and fever response [5]. In PI, a patient may not receive critical antibiotics if a fever is missed; thus, it is essential to understand if a muted fever response exists. Missing an infection in PI may lead to delayed diagnosis or treatment, which can lead to decreased quality of life as well as increased morbidity and mortality for patients [2]. Moreover, it is unclear if and how different types of PI may impact a patient’s ability to mount a fever response and baseline thermoregulation. As PIs are heterogeneous and involve different arms of the immune system, the ability of patients with PI to mount a fever response may partly depend on the underlying condition [5]. For example, patients with PI with autoinflammatory conditions, such as Familial Mediterranean Fever or familial cold autoinflammatory syndrome, are characterized by recurrent fever, whereas patients with toll-like receptor defects, such as interleukin-1 receptor-associated kinase 4 deficiency, fail to mount fever in the presence of pyogenic infections [1,5]. More information is needed to understand if individuals with PI have different body temperatures at baseline and when sick so that appropriate medical treatment can be provided in a timely manner.

There has been a recent expansion in the degree of patient involvement occurring in research studies. A recent review of 126 articles by the Patient-Centered Outcomes Research Institute in 2019 highlights how patients are being engaged as early as the study design phase in selecting study outcomes and tailoring interventions to meet patients’ needs [6]. Valuable contributions from patients have been reported in research feasibility, acceptability, rigor, and relevance by aligning the needs and concerns of patients and their clinicians. Research is deemed more meaningful for patients, with less burden and with greater adherence to interventions [6]. Efforts to involve patients in the research process are thus considered here.

### Objectives

The purpose of this study is to assess whether patients with PI exhibit lower-than-normal average body temperature compared with individuals without PI. The primary objective is to measure and compare resting body temperature at select time intervals in a cohort of individuals living with PI and unaffected controls who are adult family members without PI living in the same household. The secondary goal is to engage individuals who are affected by PI, including patients, family members, and caregivers throughout the research process as citizen scientists.

## Methods

### Overview

A prospective cohort comparison study was designed to compare 2 populations. This study was designed as a patient-stakeholder collaboration and supported by the Immune Deficiency Foundation (IDF) with oversight by the Advarra institutional review board. No outside funding was received. IDF is composed of patients with PI, along with their family, supporters, and health care professionals who work with the PI community. These stakeholders are involved in every facet of IDF and comprise the leadership, staff, board of trustees, and volunteers that enable the organization to serve the PI community in a comprehensive manner.

IDF improves the diagnosis, treatment, and quality of life of people affected by PI by fostering a community empowered by advocacy, education, and research. IDF provides accurate and timely information for patients and families living with PI and offers valuable resources. IDF sponsors education and outreach efforts for the medical community. In addition, IDF promotes, participates, and conducts research that has helped characterize PI and substantially improve treatment options. Patient needs are addressed by IDF through public policy programs and advocacy at state and federal levels.

### Objectives

The purpose of this study is to assess whether patients with PI exhibit a lower-than-normal average body temperature compared with non-PI individuals. This study tested the hypothesis that there is no difference in mean body temperature between adults diagnosed with or without PI. The primary objective is to measure and compare resting body temperature at select time intervals in a cohort of individuals living with PI and unaffected controls who are non-PI adult family members living in the same household. All subjects were given the same questionnaires, thermometers, instructions, and schedule for taking their temperatures. The secondary objective is to engage individuals living with PI throughout the entire research process as citizen scientists.

### Patient-Led Approach

This study was a patient-driven study with the participation of patients with PI from its initial inception to completion as citizen scientists. In 2017, several members of the IDF and participants in IDF online forums, such as IDF Friends and PI CONNECT, began a grassroots effort to address concerns of individuals living with PI. Although fever is considered an initial sign of infection, many individuals with PI have been reported to have a lower-than-normal average body temperature. On these online forums, patients with PI reported a temperature less than 100.4°F even when other indications of infection were present. Patients with PI expressed an interest in exploring this systematically through a patient-designed research project.

With these initial concerns, IDF participants subsequently approached IDF staff and leadership, who, in turn, contacted members of the IDF medical advisory board and PI researchers. From an online forum, a focus group at the IDF annual meeting was established to discuss concerns of patients with PI. This focus group morphed into a task force that inspired a patient-informed study. Several conference calls followed among patients with PI, IDF staff and leadership, PI clinicians, and PI researchers who expressed interest in designing and implementing a collaborative research project to assess body temperature in patients with PI. The proposed study design underwent several revisions, and a protocol was eventually agreed upon by all key stakeholders and PI representatives. Patient advocates from IDF were on the research team, which included active roles in project design, management, data collection, data analysis, and data reporting through dissemination of findings and manuscript preparation. Clinicians and researchers acted as content experts and advisors to provide input on best practices and rigorous study design, but all stakeholders agreed that the project would defer to the wishes of the patients with PI who were the ultimate drivers of the entire endeavor.

Patients with PI were involved at every step of the process. During study design, patients with PI voiced their interest among focus groups in studying differences in body temperature among healthy participants and participants with PI, which became the aim of the study. Patients with PI shared social media posts and newsletter announcements in subject recruitment and were the key participants in data collection. The materials in the study packets were generated, assembled, and mailed to participants by the IDF staff and volunteers, many of whom live with PI themselves. The study team, IDF staff, and volunteers collaboratively performed data entry, analysis, interpretation, results dissemination, and manuscript preparation. Preliminary research findings were shared with the PI community at IDF conferences and on the web as they became available.

### Recruitment

A total of 350 adults with PI were recruited from IDF rosters, and 1 adult household member without PI was also recruited per patient to serve as a control. The IDF recruited participants through direct email, newsletter announcements, and social media posts among patients with PI. All patients identified as adults aged older than 20 years in the IDF databases received a recruitment email explaining the study and linking participants to a screening questionnaire. A promotional flyer is attached in the supplementary material (Multimedia Appendix 1).

### Enrollment

The initial screening questionnaire, which was used by the IDF for subject selection, surveyed basic demographic and medical information from participants with and without PI. Inclusion criteria included adults with PI aged between 21 and 70 years, who were not acutely ill at study start and who had a willing member of his or her household without PI to serve as a comparator. Exclusion criteria included those aged below 21 years or above 70 years and those unable to take an oral temperature. Informed consent was obtained electronically from both household members of eligible participants before study enrollment. Participants who returned their signed data packets received a US $20 Amazon coupon per household as an incentive.

### Interventions

Enrolled subjects received a welcome packet with instructions, a thermometer, a data collection booklet, and return envelopes for their booklets. All participants received and used McKesson digital oral thermometers (Model 01-413BGM) to record temperatures 3 times a day for 5 consecutive days for subject convenience. The 5-day study period was chosen based on input from IDF members who believed that this time frame would be acceptable and minimally obtrusive to the community of patients with PI and family members. Each participant took his or her own temperature on arising in the morning, in the early evening, and at bedtime, at approximately the same time of day for each of the 5 days. They recorded their temperatures in a data collection booklet that was returned to the IDF at the study conclusion. Detailed instructions with pictures were provided. Participants were instructed not to drink any hot or cold fluids, smoke, eat, drink, exercise, or perform other activities that may raise or lower temperature readings at least 30 min before taking their temperature. Subjects recorded if an infection was present daily. Of note, researchers could not verify this self-reported status of infection or no infection because of the self-report nature of the study. No collateral information (such as doctor’s notes or laboratory testing) to verify this self-report was collected or analyzed in the study. After the study concluded, participants received a follow-up questionnaire to assess their overall study experience as well as their willingness to participate in similar patient-driven research in the future with the IDF.

### Statistical Analysis

Descriptive statistics were calculated. Hypothermic temperatures below 95.0°F were excluded from the analysis. The median body temperatures were compared between the 2 cohorts using the Mann-Whitney test. We also performed a subgroup analysis comparing the recorded temperatures of patients with PI and controls who self-reported being ill for each time point. Prism 6.0 (GraphPad Software) was used to perform the statistical analyses. Statistical significance was set at a *P* value <.05, and Bonferroni correction for multiple comparisons was applied.

## Results

### Participants

Of the 350 eligible households that were invited, 254 participated in the study (254/350, 72.6% participation rate). The PI and non-PI cohort demographics are summarized in Table 1. The PI population was predominantly female (218/254, 85.8%), White (248/254, 97.6%), and with a median age of 49 years. These participant demographics are similar to those of other studies from IDF [7]. The non-PI population was largely male (170/254, 66.9%), White (236/254, 92.9%), and with a median age of 53 years.

**Table 1:** Participant demographics.

Demographic		PI^a^	Non-PI
Age (years), median		49	53
**Age group (PI only; years), n**			
	21-34	53	37
	35-44	52	43
	45-54	61	64
	55-70	88	110
**Sex, n**			
	Male	35	170
	Female	218	83
	Transgender	1	1
**Race and ethnicity, n**			
	American Indian or Alaskan Native	2	1
	Asian or Pacific Islander	0	4
	Black or African American	1	1
	Hispanic or Latino	5	10
	White, non-Hispanic	248	236
	Two or more races	5	4

^a^PI: primary immunodeficiency.

### PI Diagnoses

The diagnoses of the PI cohort are summarized in Table 2. Humoral immunodeficiencies predominated with common variable immunodeficiency being the most prevalent (190/254, 74.8%). Of the total 254 cases, nonhumoral defects comprised 3 (1.2%) of the diagnoses, including chronic granulomatous disease 1 (0.4%), combined immunodeficiency 1 (0.4%), and complement deficiency 1 (0.4%).

**Table 2:** Primary immunodeficiency diagnoses.

Primary immunodeficiency diagnosis	Participants (n=254), n (%)
Common variable immunodeficiency	190 (74.8)
Hypogammaglobulinemia	32 (12.6)
Immunoglobulin G subclass deficiency	12 (4.7)
Selective Immunoglobulin A deficiency	8 (3.1)
Specific antibody deficiency	8 (3.1)
Agammaglobulinemia	1 (0.4)
Chronic granulomatous disease	1 (0.4)
Combined immunodeficiency	1 (0.4)
Complement deficiency	1 (0.4)

### Body Temperature Perceptions

We asked 254 PI respondents about their body temperature perceptions (Table 3). Of the 254 respondents, 123 (48.4%) PI respondents reported a lower-than-normal nonsick body temperature, whereas 108 (42.5%) reported a normal (between 97°F and 99°F) nonsick body temperature. When infected, 67.7% (172/254) of the PI respondents reported absence of fever with infection, whereas 19.7% (50/254) reported a normal fever response with infection. As summarized in Table 3, most participants with PI reported an abnormal nonsick body temperature when well and an absence of fever with infection when sick. These findings underscore the need to better define body temperature in patients with PI.

**Table 3:** Body temperature perceptions.

Body temperature perceptions		Frequency, n (%)
**Well condition: Which of the following statements is closest to your experiences with your day-to-day, nonsick, body temperature?**		
	My nonsick body temperature is normal (between 97°F-99°F)	108 (42.5)
	My nonsick body temperature is lower than normal (between 95.0°F-96.9°F)	123 (48.4)
	My nonsick body temperature is higher than normal (≥99.1°F)	2 (0.8)
	Not sure/don’t know	21 (8.3)
**Sick condition: When you have an infection, which of the statements below is closest to your experiences?**		
	I have a normal fever response when I get an infection	50 (19.7)
	I do not get a fever when I have an infection	172 (67.7)
	I get a very high fever when I have an infection	10 (3.9)
	Not sure/don’t know	22 (8.7)

### Body Temperature Measurements

The next step of the study focused on measuring the body temperatures of participants with PI to determine whether there were any cohort differences at baseline. During the objective measurement phase of the study, the median body temperatures for each time point on all 5 days and for the week were recorded and are summarized in Table 4.

**Table 4:** Objective median body temperatures (°F).

Time of day		PI^a^, °F (range)		Non-PI, °F (range)		*P* value	
**Daily temperatures**							
	Monday morning		97.5 (95.2-100.1)		97.2 (95.2-99.4)		.03
	Monday early evening		97.8 (95.0-99.6)		97.8 (95.1-99.3)		.81
	Monday bedtime		97.6 (95.2-100.4)		97.5 (95.2-99.8)		.28
	Tuesday morning		97.4 (95.3-99.3)		97.2 (95.4-98.9)		.002^b^
	Tuesday early evening		97.8 (95.0-99.8)		97.7 (95.4-99.8)		.06
	Tuesday bedtime		97.5 (95.4-100.5)		97.4 (95.1-100.7)		.65
	Wednesday morning		97.5 (95.0-99.6)		97.2 (95.0-99.6)		.001^b^
	Wednesday early evening		97.8 (95.3-99.7)		97.7 (95.1-99.8)		.40
	Wednesday bedtime		97.5 (95.4-99.6)		97.4 (95.0-99.6)		.67
	Thursday morning		97.4 (95.0-99.5)		97.2 (95.1-99.0)		.06
	Thursday early evening		97.8 (95.0-99.6)		97.7 (95.0-100.5)		.14
	Thursday bedtime		97.4 (95.7-99.5)		97.4 (95.0-100.6)		.32
	Friday morning		97.4 (95.2-99.5)		97.1 (95.1-101.0)		.001^b^
	Friday early evening		97.7 (95.0-99.9)		97.7 (95.5-101.2)		.71
	Friday bedtime		97.4 (95.1-99.9)		97.5 (95.0-100.9)		.91
**Weekly temperatures**							
	(Monday to Friday) morning		97.4 (95.0-100.1)		97.2 (95.0-101.0)		<.001^c^
	Early evening		97.8 (95.0-99.9)		97.7 (95.0-101.2)		.05
	Bedtime		97.5 (95.1-100.5)		97.4 (95.0-100.9)		.37

^a^PI: primary immunodeficiency.

^b^Statistically significant after Bonferroni correction of *P* value (0.05/15=0.003).

^c^Statistically significant after Bonferroni correction of *P* value (0.05/3=0.017).

### Group Comparisons

**Figure 1:**
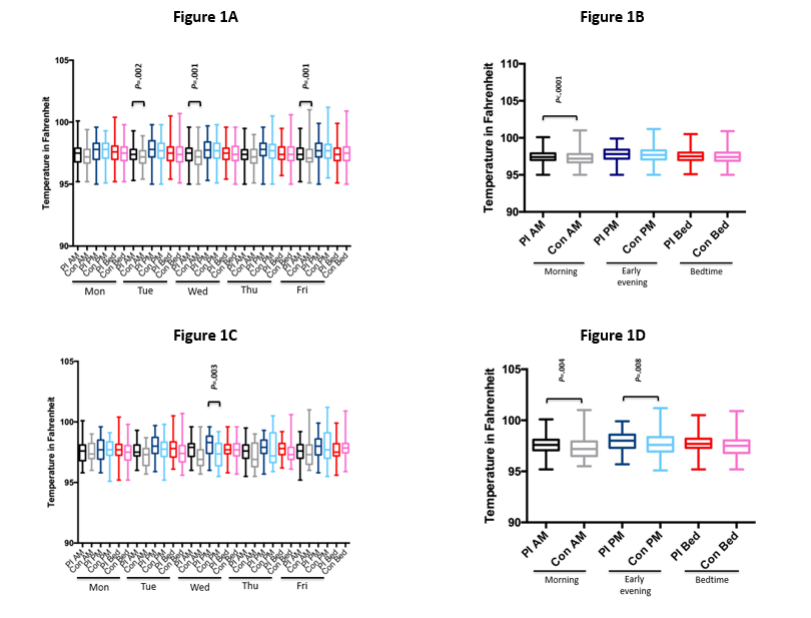
Objective median body temperatures in patients with PI and control nonprimary immunodeficiency household members. Part A depicts all daily objective body temperatures. Statistically significant *P* value (.003) after Bonferroni correction. Part B depicts all weekly objective body temperatures. Statistically significant *P* value (.017) after Bonferroni correction. Part C depicts all daily objective body temperatures when they perceive an infection. Statistically significant *P* value (.003) after Bonferroni correction. Part D depicts all weekly objective body temperatures when sick. Statistically significant *P* value (.017) after Bonferroni correction. Con: control; PI: primary immunodeficiency.

Figure 1 graphically shows that compared with controls without PI, individuals with PI had minimally higher median body temperatures in the morning, but not early evening or bedtime, on 3 of 5 days (Tuesday, Wednesday, and Friday) in the top left panel. In the top right panel, Figure 1 further demonstrates that PI subjects had a minimally higher median temperature in the morning during the study.

To examine if these differences in body temperature varied with infection, we compared the temperatures of each cohort during the time of a self-reported illness. Respondents were asked if they perceived themselves to be sick with an infection during each day of the study, but the study design did not permit us to corroborate respondent self-report with an objective assessment such as laboratory testing or physician examination. To assess how subjects’ self-reported perception of being sick matched with their recorded temperatures, we tabulated their subjective responses with their recorded temperatures in Tables 5 and 6 for subjects with PI and no PI, respectively. Table 5 shows that 22.4% (range 20.9%-23.6%) of PI subjects self-reported being sick at some point during the study period and that fever with temperature ≥100.4°F only occurred twice. Moreover, 77.2% (range 64.4%-87.9%) reported having a normal temperature (between 97°F and 99°F), whereas 16.2% (range 6.8%-28.8%) reported having a lower-than-normal temperature (between 95.0°F and 96.9°F) when sick.

**Table 5:** Frequency of reported body temperature when sick among primary immunodeficiency subjects.

Time of day	Primary immunodeficiency respondents		Temperature (°F), n (%)					
	Number of respondents	Percentage of respondents who reported being sick, n (%)^a^		≥100.4	99.1-100.3	97.0-99.0	95.0-96.9	Did not record
Monday morning	59	59 (23.2)		0 (0)	2 (3.4)	40 (67.8)	17 (28.8)	0 (0)
Monday early evening	59	59 (23.2)		0 (0)	6 (10.2)	38 (64.4)	15 (25.4)	0 (0)
Monday bedtime	59	56 (22.0)		1 (1.7)	1 (1.7)	50 (84.7)	4 (6.8)	0 (0)
Tuesday morning	55	55 (21.7)		0 (0)	2 (3.6)	44 (80.0)	9 (16.4)	0 (0)
Tuesday early evening	55	54 (21.3)		0 (0)	5 (9.3)	42 (77.8)	7 (13.0)	1 (1.9)
Tuesday bedtime	55	53 (20.9)		1 (1.9)	1 (1.9)	39 (73.6)	12 (22.6)	2 (3.8)
Wednesday morning	60	60 (23.6)		0 (0)	3 (5.0)	48 (80.0)	9 (15.0)	0 (0)
Wednesday early evening	60	58 (22.8)		0 (0)	8 (13.8)	43 (74.1)	7 (12.1)	2 (3.4)
Wednesday bedtime	60	58 (22.8)		0 (0)	2 (3.4)	48 (82.8)	8 (13.8)	2 (3.4)
Thursday morning	58	58 (22.8)		0 (0)	2 (3.4)	43 (74.1)	13 (22.4)	0 (0)
Thursday early evening	58	55 (21.7)		0 (0)	3 (5.5)	48 (87.3)	4 (7.3)	3 (5.5)
Thursday bedtime	58	58 (22.8)		0 (0)	1 (1.7)	51 (87.9)	6 (10.3)	0 (0)
Friday morning	58	58 (22.8)		0 (0)	1 (1.7)	45 (77.6)	11 (19.0)	1 (1.7)
Friday early evening	58	57 (22.4)		0 (0)	5 (8.8)	42 (73.7)	8 (14.0)	2 (3.5)
Friday bedtime	58	55 (21.7)		0 (0)	4 (7.3)	40 (72.7)	9 (16.4)	4 (7.3)
Mean^b^ (%)	N/A^c^	22.4		0.2	5.4	77.2	16.2	2.0

^a^Percent sick is based on 254 respondents. For each temperature, percentage is based on the number of sick.

^b^Means are the average of all percentages per column.

^c^N/A: not applicable.

Table 6 shows that 6.3% (range 3.9%-8.7%) of subjects without PI self-reported being sick at some point during the study period and that fever only occurred 7 times. Furthermore, 60.9% (range 42.1%-80.0%) reported having a normal temperature, whereas 30.8% (range 10.0%-53.8%) reported having a lower-than-normal temperature when sick.

**Table 6:** Frequency of reported body temperature when sick among subjects with no primary immunodeficiency.

Time of day	Respondents with no primary immunodeficiency		Temperature (°F), n (%)				
	Number of respondents	Percentage of respondents who reported being sick, n (%)^a^	≥100.4	99.1-100.3	97.0-99.0	95.0-96.9	Did not record
Monday morning	17	17 (6.7)	0 (0)	0 (0)	13 (76.5)	4 (23.5)	0 (0)
Monday early evening	17	17 (6.7)	0 (0)	1 (6.9)	13 (76.5)	3 (17.6)	0 (0)
Monday bedtime	17	17 (6.7)	0 (0)	1 (5.9)	11 (64.7)	5 (29.4)	0 (0)
Tuesday morning	22	22 (8.7)	0 (0)	0 (0)	13 (59.1)	9 (40.9)	0 (0)
Tuesday early evening	22	22 (8.7)	0 (0)	2 (9.1)	16 (72.7)	4 (18.2)	0 (0)
Tuesday bedtime	22	21 (8.3)	1 (4.8)	0 (0)	11 (52.4)	9 (42.9)	1 (4.8)
Wednesday morning	19	19 (7.5)	0 (0)	1 (5.3)	8 (42.1)	10 (52.6)	0 (0)
Wednesday early evening	19	18 (7.1)	0 (0)	1 (5.6)	10 (55.6)	7 (38.9)	1 (5.6)
Wednesday bedtime	19	19 (7.5)	0 (0)	2 (10.5)	13 (68.4)	4 (21.1)	0 (0)
Thursday morning	13	13 (5.1)	0 (0)	0 (0)	6 (46.2)	7 (53.8)	0 (0)
Thursday early evening	13	13 (5.1)	1 (7.7)	2 (15.4)	6 (46.2)	4 (30.8)	0 (0)
Thursday bedtime	13	11 (4.3)	1 (9.1)	0 (0)	7 (63.6)	3 (27.3)	2 (18.2)
Friday morning	11	11 (4.3)	1 (9.1)	0 (0)	6 (54.5)	4 (36.4)	0 (0)
Friday early evening	11	11 (4.3)	2 (18.2)	1 (9.1)	6 (54.5)	2 (18.2)	0 (0)
Friday bedtime	11	10 (3.9)	1 (10.0)	0 (0)	8 (80.0)	1 (10.0)	0 (0)
Mean^b^ (%)	N/A^c^	6.3	3.9	4.5	60.9	30.8	1.9

^a^Percent sick is based on 254 respondents. For each temperature, percentage is based on the number that is sick.

^b^Means are the average of all percentages per column.

^c^N/A: not applicable.

For those patients and controls who reported being sick, the median temperatures at each time of day are tabulated in Table 7.

Figure 1 shows that individuals with PI who self-reported being sick had minimally higher median body temperatures in the early evening midweek on Wednesday in the bottom left panel. The bottom right panel in Figure 1 further demonstrates that the same PI subjects had minimally higher median temperatures in the morning and early evening in the bottom panel during the study. Overall, we found the majority of participants, regardless of PI status, had normal measured temperatures during times of reported infection, and that fevers were rare.

**Table 7:** Objective median body temperatures (°F) when sick.

Time of day		PI^a^, °F (range)	Non-PI, °F (range)	*P* value
**Daily temperatures**				
	Monday morning	97.6 (95.8-100.1)	97.3 (96.0-99.0)	.99
	Monday early evening	97.7 (95.8-99.6)	97.7 (95.1-99.1)	.87
	Monday bedtime	97.7 (95.2-100.4)	97.5 (95.2-99.8)	.17
	Tuesday morning	97.5 (96.0-99.3)	97.3 (95.7-98.7)	.09
	Tuesday early evening	98.0 (95.9-99.7)	97.8 (95.2-99.8)	.14
	Tuesday bedtime	97.8 (96.1-100.5)	97.4 (95.6-100.7)	.11
	Wednesday morning	97.9 (96.0-99.6)	96.9 (95.7-99.6)	.01
	Wednesday early evening	98.3 (96.0-99.6)	97.4 (95.5-99.2)	.003^b^
	Wednesday bedtime	97.7 (95.8-99.6)	97.7 (95.7-99.6)	.54
	Thursday morning	97.6 (95.5-99.5)	96.9 (95.5-99.0)	.29
	Thursday early evening	97.9 (95.7-99.3)	97.2 (95.9-100.5)	.18
	Thursday bedtime	97.8 (96.2-99.2)	97.3 (96.1-100.6)	.10
	Friday morning	97.6 (95.2-99.2)	97.3 (96.0-101.0)	.48
	Friday early evening	98.0 (95.8-99.9)	97.7 (95.5-101.2)	.92
	Friday bedtime	97.5 (95.6-99.9)	97.9 (95.9-100.9)	.39
**Weekly temperatures**				
	(Monday-Friday) morning	97.6 (95.2-100.1)	97.2 (95.5-101.0)	.004^c^
	Early evening	98.0 (95.7-99.9)	97.6 (95.1-101.2)	.008^c^
	Bedtime	97.7 (95.2-100.5)	97.5 (95.2-100.9)	.04

^a^PI: primary immunodeficiency.

^b^Statistically significant with Bonferroni adjusted *P* value (0.05/15=.003).

^c^Statistically significant with Bonferroni adjusted *P* value (0.05/3=.017).

### Poststudy Patient Experience Survey

The research team developed a postassessment survey to obtain participant feedback because this was the first time that IDF used such a citizen science approach to research. A total of 67 participants (67/254, 26.4% participation rate) completed the poststudy assessment. Of the 67 participants, a total of 65 (97%) respondents were participants with PI. Overall, the respondents appeared to have a positive experience with this research endeavor: (1) 94% (63/67) reported that it would be *very likely* for them to read a summary report of the study when posted on IDF’s website; (2) 81% (54/67) reported it would be *very likely* that they would read a published peer-reviewed article; and (3) 91% (61/67) indicated that it would also be *very likely* that they would participate in future IDF research studies, although 93% (62/67) of participants had never taken part in previous IDF research studies. Overall, participants were enthusiastic about the research process that was participant-driven at every step. Members of the PI community, including those not directly involved in the research, were very engaged when preliminary study results were presented at IDF meetings. Members of the research team and other key stakeholders anecdotally reported witnessing and participating in many interesting conversations about the findings at various IDF events and online forums. Highlighting the overwhelming positive response to the project and the expectation that this work would be shared outside the IDF community are among the driving forces behind publishing this manuscript.

## Discussion

### Principal Findings

Although discrepancies between subjective and objective core body temperatures in chronic disease have been reported previously, limited literature exists on average body temperature in persons with PI [3,8]. Hamilos et al [8] monitored continuous 24-hour body temperature recordings of 7 patients with chronic fatigue syndrome (CFS) and compared them against 3 sets of age-, sex-, and weight-matched cohorts (normal controls, subjects with seasonal allergy, and subjects with major depression). Despite frequent self-reports of subnormal body temperature and low-grade fever, CFS subjects were found to have normal core body temperatures [8]. To our knowledge, our study is the first to evaluate average body temperature in PI subjects, thus improving our understanding of another chronic disease and addressing an important knowledge gap.

Interestingly, our study did not corroborate the beliefs of patients with PI and their caregivers regarding their temperatures when ill and in their usual state of health. Many caregivers and persons living with PI believe that patients with PI run lower-than-normal sick and nonsick temperatures. Of the 254 participants, 123 (48.4%) participants subjectively reported this, whereas 108 (42.5%) reported normal nonsick temperatures. Of the 254 participants, 172 (67.7%) respondents subjectively reported an absence of fever during infection, whereas 50 (19.7%) reported fever with infections. Our findings suggest that patients with PI appear to have minimally higher morning temperatures compared with controls even after adjusting for multiple comparisons [9]. However, these results need to be interpreted with caution, given our small sample size and methodological study design issues. Less than 25.0% of the subjects with PI self-reported being sick at some point during the study period. Of these, the majority (196/254, 77.2%) reported a normal temperature, (42/254, 16.4%) had a lower-than-normal temperature, and (1/254, 0.2%) had a fever. Less than 7.0% of the subjects with no PI self-reported being sick at some point during the study period. Of these, most (155/254, 60.9%) reported a normal temperature, whereas (78/254, 30.8%) had a lower-than-normal temperature and (10/254, 3.9%) had a fever. Our findings suggest that patients with PI may have minimally higher morning and early evening median temperatures compared with healthy controls when subjects self-report being sick. Such small differences fall within normal variation for daily temperatures and are likely not clinically meaningful.

Although we found statistically significant differences in body temperatures between subjects with PI and no PI when they self-reported being sick or healthy, the clinical significance of such small differences is unclear and should be interpreted cautiously. Previous studies have shown variations in thermoregulation among the general population. An observational cohort study of 35,488 patients (mean age 52.9 years, 64% women, 41% non-Whites) from a large academic hospital from 2009 to 2014 showed that of 243,506 outpatient temperature measurements, the mean temperature was 36.6°C (97.9°F) with a 95% CI of 35.7°C-37.3°C (96.3°F-99.1°F; [10]). Older individuals were the coolest (−0.021°C for every decade; *P*<.001), and African American women were warmer than White men (+0.052°C; *P*<.001). Several comorbidities were linked to lower temperatures, including hypothyroidism (−0.013°C; *P*=.01) as well as higher temperatures including cancer (+0.020; *P*<.001) and BMI (+0.002 per kg/m^2^; *P*<.001). Measured factors explained only 8.2% of individual temperature variation, whereas unexplained temperature variation was a significant predictor of subsequent mortality: controlling for all measured factors, an increase of 0.149°C was linked to 8.4% higher 1-year mortality (*P*=.02; [10]). Possible explanations for higher median body temperatures in patients with PI, which should be considered in future studies, include differences in subclinical infection/inflammation, hormone levels, thyroid function, comorbidities including malignancy, dietary intake and activity level, and body composition.

As many of our subject with PI were women of childbearing age, future studies are needed to elucidate the potential roles of hormonal changes and the menstrual cycle on body temperature, given this major difference in sex between our study and control populations. A study of core temperatures in young, healthy women with regular menstrual cycles and baseline fluctuations of >0.5°C in basal core temperature during luteal and follicular phases revealed consistently higher temperatures in the luteal phase than in the follicular phase [11]. The small variation in temperature between PI and non-PI participants in this study may be related to hormonal effects causing fluctuating body temperatures in menstruating females, so future studies of body temperature differences should account for menstrual cycles, especially if large sex differences in study populations exist, as is the case in this study.

It is also possible that the fever response fundamentally differs between subjects with and without PI, and future studies are needed to elucidate the involved immunocytes and cytokine milieu of fever in patients with PI [12]. PIs are diverse, and the arms of the immune system that are affected in these heterogeneous conditions likely differentially impact thermoregulation and the ability to mount a fever response. Future studies are needed to assess whether this difference in fever response is a manifestation of immune dysregulation among patients with PI.

### Study Strengths

Our study had several strengths, including the prospective study design, and continued collaboration from the community from initial inception to study completion. This commitment from the PI community enabled the study to be done in partnership with participants at all stages of the endeavor, which facilitated high participation and engagement. The experience of working with citizen scientists was positive for collaborators as well. There was a conscious decision by all stakeholders that we would always err on following the wishes of the participants who were the driving force behind the project. The innovative, patient-driven, and team-based approach to this study was well received by the PI community, as seen in the poststudy assessment as well as collaborators. The research team and the IDF staff will continue to build upon this experience by adopting this paradigm of actively engaging persons living with PI at all stages of research endeavors in future projects.

### Limitations

However, we acknowledge that our study has several limitations, such as differences in household settings and individual differences in taking a temperature. As participants were not observed directly, we cannot exclude the possibility that temperatures may have been taken inconsistently or in slightly different household settings with different thermostat settings. Providing the same thermometer and instructions partially mitigates this concern. In addition, we acknowledge that our analyses do not control for daily medications (eg, antipyretics), activity, or subclinical illness symptoms. Notably, our study did not assess the impact of immunomodulator use, such as steroids or biologics, which can interfere with fever response. Infections were self-reported by patients and not verified by providers or objective collateral information, which limits data interpretation. A major limitation of this study is that infection was not defined explicitly to participants during the study period, which vastly limits the ability to draw definitive conclusions about body temperature differences during times of illness in this study. Because these limitations potentially seriously compromise the scientific integrity and validity of our study, all stakeholders participated in extensive discussions with content experts in study design, statistics, and immunology, regarding these weaknesses during the inception and planning stages. Ultimately, stakeholders jointly decided to err on the side of following what patients with PI stated that they could and were willing to do for the study.

Our study was also not designed to assess the impact of the *wearing off effect* that some patients with humoral immunodeficiency on monthly intravenous immunoglobulin G (IgG) infusion can experience before their next dose [7,13,14]. The wearing off effect is associated with decreased treatment efficacy, increased infection susceptibility, and diminished quality of life. This effect should be considered since a 2003 IDF study consisting of 1186 subjects showed that 308 (25.96%) of patients with PI reported feeling wearing off occasionally, whereas 498 (41.99%) reported wearing off as a typical experience of their therapy. Patients experiencing a wearing off effect can benefit with more frequent dosing (every 3 vs 4 weeks) or with switching to a subcutaneous route [15]. Future studies are needed to determine how body temperature among patients with PI might be influenced by this phenomenon and whether the IgG replacement route plays a role.

In addition, our study used a convenience sample without randomization. We cannot exclude the possibility of participation bias among different types of patients with PI with respect to diagnosis or other demographic factors. Subjects were not well balanced in the type of PI or gender; thus, our findings need to be interpreted cautiously. However, as humoral immunodeficiencies account for most PIs, with common variable immune deficiency being the most common, it is not surprising that our cohort is skewed this way. Our study design cannot assess the potential effect of hormones, sex, or age on the outcomes of interest. Future studies specifically including men, children, young adults, and older adults with PI and appropriate controls that are matched by age and sex are needed.

Online forums pose unique challenges for patient-led studies. There is a unique non–face-to-face platform for interactions and the possibility of unpredictable security issues that may complicate informed consent. An interdisciplinary team, including team members with expertise in computing and ethics, may be important for troubleshooting such difficulties. Special considerations such as whether a forum should be facilitated or moderated, an informed consent process that is not done in person, language use, and data analysis are all factors considered in previous studies of online forums [16]. Considering how the start of this study began on an online forum, it is important to pay attention to these factors in future studies.

### Poststudy Survey

Our poststudy questionnaire encouraged feedback from the PI community after study completion. An important caveat is that the questionnaire only had a 26.4% (67/254) participation rate. Nonetheless, this rate is comparable with that reported in other studies conducted by IDF. Although many respondents were first-time participants in IDF research, the patient experience was largely positive. Participants were engaged in the study, and 91% (61/67) reported that they would most likely participate in future IDF research studies. Most participants were also interested in reading study results, which we are enthusiastic to share. Closing the loop with a poststudy questionnaire highlights the patient-driven approach of this study and underscores the investment of the target population in research that directly benefits their community.

### Conclusions

This study highlights that individuals with PI are knowledgeable about their conditions and can offer unique insights and direction to researchers. Similarly, this study also demonstrates that collaboration with patient advocacy groups may facilitate high participation among the target population, giving new meaning to the concept of patient-centered and patient-driven research for future studies. We acknowledge that our study has several methodological shortcomings and did not clearly resolve the original research question posed by the PI community. Nonetheless, this endeavor demonstrates that the PI community has the desire and ability to conceive, design, and implement citizen science when given the support to do so.

## Appendix

Multimedia Appendix 1 Promotional flyer for the Immune Deficiency Foundation fever study.

## Abbreviations

CFS: chronic fatigue syndrome
IDF: Immune Deficiency Foundation
PI: primary immunodeficiency
