# Supplementary material for: Persons Living With Primary Immunodeficiency Act as Citizen Scientists and Launch Prospective Cohort Body Temperature Study
Source: J Particip Med. 2020 Nov 30;12(4):e22297. doi: 10.2196/22297 (PMC7735893; doi:10.2196/22297)
Supplement: Multimedia Appendix 1 [file jopm_v12i4e22297_app1.docx]

**Participate in IDF Fever Study**


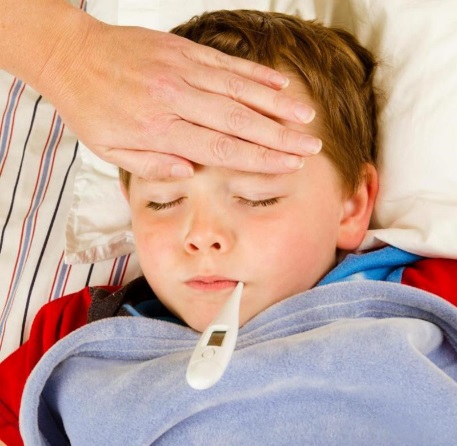
IDF has an exciting research project that developed from discussions in the IDF PI CONNECT Research Forum and IDF Friends. Many individuals with PI report having a lower than normal average body temperature. As a result, when they do have signs of an infection, frequently their temperature does not rise to 100.4°F, the threshold upon which doctors begin to prescribe antibiotics. If this occurs, patients who have infections may not receive critical antibiotics due to missed signs of fever.

These conversations about fever in PI even made their way into the PI CONNECT Live! session held at the IDF 2015 National Conference. Here, the idea for a patient-based study on fever in PI was born.

Currently, no known literature exists on average body temperature in persons with PI. In order to address this gap in the literature, IDF would like to conduct a study to determine if people with PI have lower than average body temperature. This study will be 100% independently funded by IDF through funds raised through IDF Walk for Primary Immunodeficiency.

We are on our way to making this study a reality, but we need your participation! In the next month or so, please be on the lookout for an e-mail invitation from us to take a brief, online screening survey.

Here is what we are looking for:

- Households where there is at least one adult, 21 years of age or older, who has a PI AND another non-PI adult in the household who is willing to participate in the study.

What does the study entail?

- Both the person with PI and the other non-PI adult in the household will be asked to take their oral temperature three times per day, five days in row and record it in a paper log-book provided to you by IDF.
- Each day we will also ask you and the other adult in the house to tell us about what medications, supplements or therapies you might be taking that day and ask about how you feel in general.
- At the end of the study, you will be asked to return the log-book to IDF in a pre-paid envelope provided by IDF.
- IDF will provide you with a digital-oral thermometer for the study that you can keep.
- Those households that participate in the actual study will each receive a $20 e-gift card that can be used on Amazon.com (one per household).

Although not everyone may be selected to participate in the study, this survey is an important first-step.

**Please be on the lookout for the survey invitation in your e-mail.**
